# Effect of transcutaneous electrical acupoint stimulation on pregnancy outcomes in women with *in vitro* fertilization-embryo transfer: A systematic review and meta-analysis

**DOI:** 10.3389/fcell.2022.1068894

**Published:** 2022-12-12

**Authors:** Fengya Zhu, Bo Zhao, Jie Wu, Shao Yin, Tingting Ma, Zimeng Li, Xinyun Zhu, Tianyu Wang, Bin Yang, Deya Che

**Affiliations:** ^1^ Acupuncture and Tuina School, Chengdu University of Traditional Chinese Medicine, Chengdu, China; ^2^ Traditional Chinese Medicine Department, Zigong First People’s Hospital, Zigong, China; ^3^ Hospital of Chengdu University of Traditional Chinese Medicine, Chengdu, China; ^4^ The Third People's Hospital of Chengdu, Chengdu, China; ^5^ People's Hospital of Leshan, Leshan, China

**Keywords:** transcutaneous electrical acupoint stimulation (TEAS), *in vitro* fertilization-embryo transfer (IVF-ET), pregnancy outcomes, systematic review, meta-analysis

## Abstract

**Objective:** The purpose of this systematic review and meta-analysis was to evaluate the efficacy and safety of transcutaneous electrical acupoint stimulation (TEAS) on pregnancy outcomes in women undergoing *in vitro* fertilization-embryo transfer (IVF-ET), in order to provide evidence-based medical support.

**Methods:** We searched the Cochrane Library, Embase, PubMed, Web of Science, SinoMed, and CNKI for relevant randomized controlled trials (RCTs) from inception to 31 May 2022, using the search terms “transcutaneous electrical acupoint stimulation,” “TEAS,” “*in vitro* fertilization-embryo transfer,” “IVF-ET,” “randomized controlled trial,” and “clinical trials.” The experimental group was treated with TEAS or combined with ovulation-inducing medication, and the control group was treated with mock TEAS (mTEAS), ovulation-inducing medication, or no intervention. The main outcome was the clinical pregnancy rate. Secondary outcomes were the embryo implantation rate, live birth rate, biochemical pregnancy rate, and number of oocytes retrieved. Stata15.1 software was used for data summary and analysis.

**Results:** This review involved 15 RCTs and 4,281 participants. TEAS were superior to the control group for improving the clinical pregnancy rate [RR: 1.29, 95% CI: 1.19 to 1.40; *p* < 0.001; I^2^ = 23.0%], embryo implantation rate [RR: 1.43, 95% CI: 1.22 to 1.69; *p* < 0.001; I^2^ = 35.9%], live birth rate [RR: 1.33, 95% CI: 1.14 to 1.54; *p* < 0.001; I^2^ = 47.3%], and biochemical pregnancy rate [RR: 1.15, 95% CI: 1.05 to 1.26; *p* = 0.003; I^2^ = 49.1%], without significant heterogeneity. TEAS had no statistically significant effect on the number of oocytes retrieved as compared with the control group, and the heterogeneity was high [SMD: 0.34, 95% CI: -0.04 to 0.72; *p* = 0.081; I^2^ = 77.6%]. We performed subgroup analysis based on the sample size, interventions and intervention time-point. The results showed that the sample size had no effect on the results. There was no significant difference between TEAS and ovulation-inducing medication in the clinical pregnancy rate or the embryo implantation rate. In addition, TEAS did not significantly increase the embryo implantation rate or the live birth rate, compared with no intervention. In terms of safety, mild allergic symptoms were found in both the experimental group and the control group.

**Conclusion:** In general, existing evidence supports the potential value of TEAS as an adjunctive treatment for improving pregnancy outcomes. High-quality, large-sample RCTs are needed to further support this conclusion.

**Systematic Review Registration:** https://www.crd.york.ac.uk/PROSPERO/display_record.php?RecordID=334892, identifier PROSPERO CRD42022334892.

## 1 Introduction

Fertility is the primary factor in reproduction; however, infertility has been recognized by the World Health Organization (WHO) as a worldwide public health problem (Macaluso et al., 2010). Infertility is defined as “historically defined by the failure to achieve a successful pregnancy after 12 months or more of regular, unprotected sexual intercourse or due to an impairment of a person’s capacity to reproduce either as an individual or with her/his partner.” (Definitions of infertility and recurrent, 2020). The global prevalence of infertility is approximately 9% (Boivin et al., 2007), but in the UK, this figure exceeds 12.5% (Datta et al., 2016). In all, 56% of infertile couples are seeking medical help, but fewer than 25% of infertile patients have received professional medical treatment (Boivin et al., 2007). Infertility inflicts great psychological distress on women (Watts and Faraj, 1990; Cousineau and Domar, 2007) and can cause mental disorders (Sbaragli et al., 2008; Volgsten et al., 2008) and even suicide (Kjaer et al., 2011).

With the rapid development of assisted reproductive technology (ART), some reproductive problems have been effectively solved (Bjelica and Nikolić, 2015). *In vitro* fertilization-embryo transfer (IVF-ET)-based ART is performed with high frequency worldwide. However, for most women in developing countries, infertility services cannot be popularized due to the unequal distribution of medical resources. The cost is also unaffordable for many (Bell, 2010). At the same time, due to regional cultural and ideological differences, infertile women face increased social discrimination (Cui, 2010). More importantly, the clinical pregnancy rate of IVF-ET is only 30%–40% (Kupka et al., 2016; De Geyter et al., 2018). Therefore, improving the clinical pregnancy rate of IVF-ET is still a major challenge in the field of reproduction.

Transcutaneous electrical acupoint stimulation (TEAS) is a type of mixed therapy developed on the basis of the combination of percutaneous nerve electrical stimulation and traditional acupuncture. Compared to traditional acupuncture, it is a non-invasive, painless treatment technique (Hsu et al., 2017). TEAS has been widely used in reproductive medicine in recent years (Qu et al., 2017a). Multiple studies have shown that TEAS significantly improves the clinical pregnancy rate, embryo implantation rate, and live birth rate (Zhang et al., 2011; Shuai et al., 2019). It can also increase the patient’s basic endocrine level and endometrial receptivity, as well as the number and quality of embryos (Shuai et al., 2015; Zheng et al., 2015). However, another study pointed out that TEAS had no statistically significant effect on the rate of high-quality embryos or that of clinical pregnancy (Zhai et al., 2022). More importantly, we found that the details of clinical intervention for TEAS were different, and whether different intervention time-point have an effect on clinical pregnancy outcomes needs to be further explored.

Therefore, we conducted a systematic review and meta-analysis of published randomized controlled trials (RCTs) using the PRISMA (Preferred Reporting Items for Systematic Reviews and Meta-Analyses) guidelines (Moher et al., 2009), aiming to evaluate the efficacy and safety of TEAS for IVF-ET pregnancy outcomes, with a view to providing evidence-based medical support.

## 2 Methods

### 2.1 Eligibility criteria

Only RCTs published in journals on the topic of TEAS for IVF-ET pregnancy outcomes were included in this review. The experimental group was treated with TEAS, alone or combined with ovulation-inducing medication, and the control group was treated with mock TEAS (mTEAS), ovulation-inducing medication, or no intervention. The main outcome was the clinical pregnancy rate. Secondary outcomes were the embryo implantation rate, live birth rate, biochemical pregnancy rate, and number of oocytes retrieved.

Clinical pregnancy is defined as detection of an intrauterine gestational sac with fetal heartbeat 4–5 weeks after embryo transfer by transvaginal ultrasound scan, and the clinical pregnancy rate = (clinical pregnancy cases/number of participants in each group) × 100%. Live birth is defined as the delivery of a live infant after 28 weeks of gestation, and the live birth rate = (number of live births/number of participants in each group) × 100%. Biochemical pregnancy is defined as serum positivity for β -human chorionic gonadotropin (β-HCG >5 U/L) 2 weeks after embryo transfer, and the biochemical pregnancy rate = (biochemical pregnancy cases/number of participants in each group) × 100%. The embryo implantation rate = (number of embryos/number of embryos transferred) × 100%.

The following were excluded from the analysis: a) conference papers, comments, reviews, animal experiments, retrospective studies, case-control studies, etc.; b) studies directly comparing TEAS with different frequencies, waveforms, and intervention times; and c) studies with outcomes that did not include the clinical pregnancy rate.

### 2.2 Search strategy

Embase, PubMed, the Cochrane Library, Web of Science, SinoMed, and CNKI were searched. Relevant randomized controlled trials were obtained from inception to 31 May 2022. The search terms included “transcutaneous electrical acupoint stimulation,” “TEAS,” “*in vitro* fertilization-embryo transfer,” “IVF-ET,” “randomized controlled trial,” and “clinical trials.” We conducted a manual search of relevant references to identify other potentially eligible studies. Grey literature and data results on the research registry platforms were not considered because we did not have access to them. The detailed search strategy is outlined in Supplementary Materials S1.

### 2.3 Study selection

Two reviewers searched for and screened potential articles according to the retrieval strategy, using EndNote X9 and further manual procedures to remove duplicates. After duplication removal, articles were screened by title and abstract. The two reviewers performed full-text evaluation according to the inclusion criteria to finalize the eligible articles. Any disagreements were resolved by discussion between the two reviewers, and if no agreement was reached, a decision was adjudicated by a third reviewer.

### 2.4 Data extraction

Using a standardized form formulated in advance, two reviewers independently extracted data from the eligible articles. The main contents included the first author, publication year, language, sample size, age, intervention details, results, and adverse events. All the above were agreed upon after cross-checking by the two reviewers, and any disagreements were resolved by a third reviewer.

### 2.5 Assessment of risk of bias

Two reviewers used the Cochrane Collaboration tool to assess the risk of bias (ROB) (Higgins et al., 2011) for each RCT. The evaluation included random sequence generation, allocation concealment, blinding of participants and personnel, blinding of outcome assessment, incomplete outcome data, selective reporting, and other biases. The risk level of each area was rated as low, high, or unclear. Any disagreement was resolved by discussion between two people. Any remaining differences were resolved by a third reviewer.

### 2.6 Data synthesis and statistical analysis

Stata15.1 software was used for statistical analysis. Risk ratios (RR) and 95% confidence intervals (CI) were used for dichotomous variables and continuous data were presented as standardized mean differences (SMD) with 95% CI. *p* < 0.05 indicated statistical significance. If there was significant heterogeneity, the random-effects model was used (I^2^ < 50% or *p* > 0.05). Subgroup analysis was performed according to the pre-determined subgroups to explore the source of heterogeneity, and sensitivity analysis was conducted to verify the stability of the results. Finally, publication bias was evaluated by the Begg and Egger tests. If there was an obvious publication bias, we also used the trim-and-fill method to verify the stability of the results (Duval and Tweedie, 2000).

### 2.7 Assessment of evidence quality

Grading of Recommendations Assessment, Development, and Evaluations (GRADE) (Schünemann et al., 2008) was used to assess the quality of evidence. It includes risk of bias, indirectness, inconsistency, inaccuracy, and publication bias. The quality of evidence was divided into high, moderate, low, and very low levels. All evaluations were conducted independently by two reviewers, with unresolved differences determined by a third reviewer.

## 3 Results

### 3.1 Search results

**FIGURE 1:**
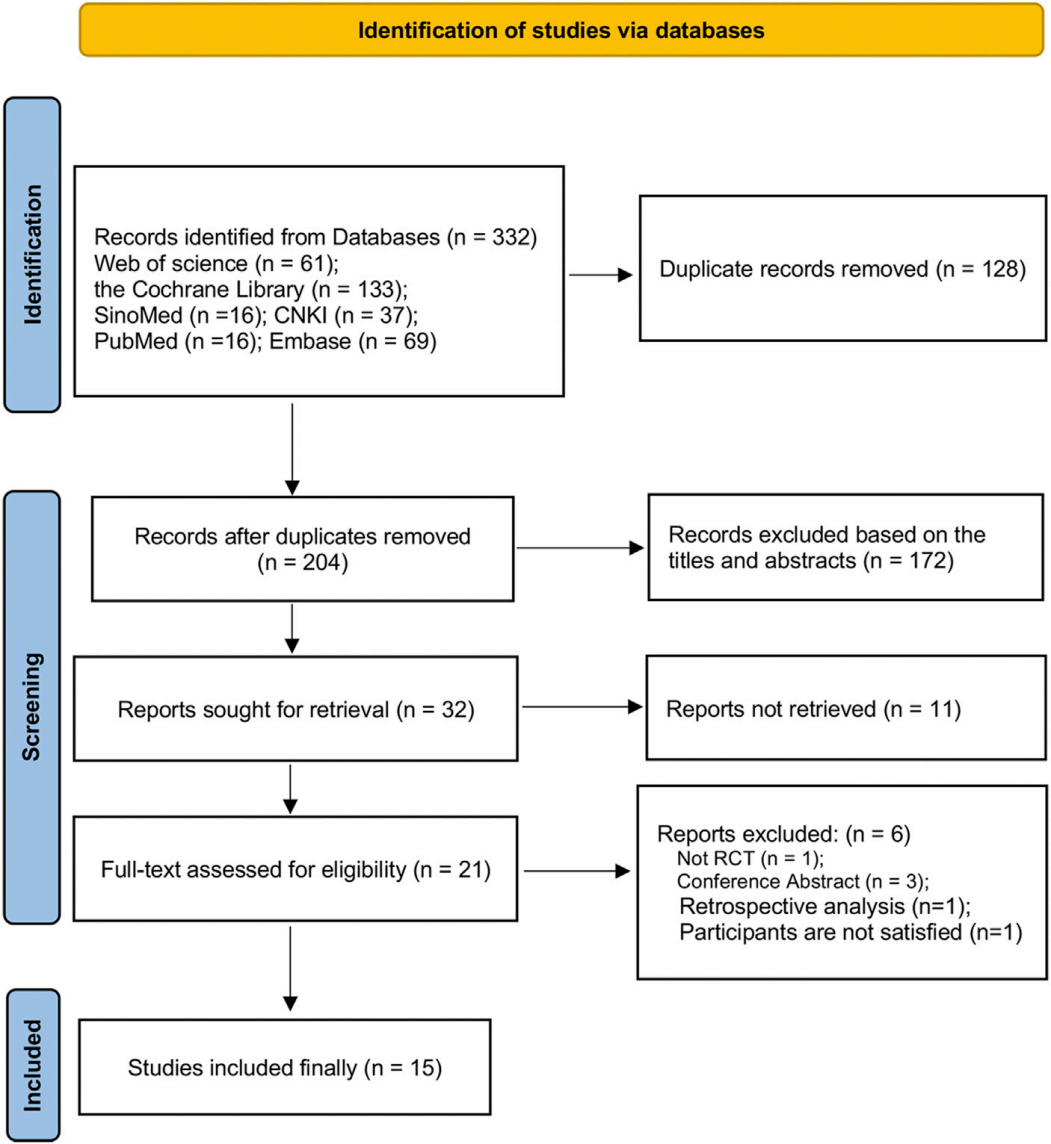
The screening flowchart.

In total, 332 studies were preliminarily retrieved from six databases. After removing duplicates, we screened them again according to titles and abstracts. Finally, 15 eligible RCTs (Zhang et al., 2011; Fang et al., 2014; Xu et al., 2014; Shuai et al., 2015; Zheng et al., 2015; Zhong et al., 2016; Qu et al., 2017b; Zhong and Zhang, 2017; Dong et al., 2018; Zhang and Zhong, 2018; Shuai et al., 2019; Zhao and Fang, 2019; Zhenhong and Yang, 2019; Zhou et al., 2021; Zhai et al., 2022) were obtained after reading of the full text. The specific screening flowchart is shown in Figure 1. See Supplementary Materials for the exclusion list and reasons for the full-text assessment stage.

### 3.2 Characteristics of included studies

A total of 15 RCTs (4,281 participants), published between 2011 and 2022, mainly in Chinese or English, with a minimum sample size of 60 participants and a maximum of 1,761 participants, were included in this review. TEAS was applied in the experimental groups, but the frequency, duration, and course of the intervention differed. The control group was mTEAS, ovulation-inducing medication, or no intervention. Each RCT differed in the selection of acupoints. Only one RCT (19) reported adverse events, and mild allergic symptoms were observed in both groups. Detailed characteristics are shown in Table 1.

**TABLE 1:** Characteristics of included studies.

Included studies	Language	Sample size (I/C)	Age [y, mean (SD)] (I/C)	Intervention	Comparison	Acupuncture points	Adverse events (I/C)	Outcomes
Zhang et al, (2011)	English	100/99	32.6 (4.9)/31.5 (5.2)	TEAS (24 h before ET and 30min after ET, 2 Hz, 15–20 mA, and 30 min)	mock TEAS (30 min after ET, intermittent 2 Hz, i5mA, and 30 min)	Diji (SP8)	Not reported	①②③④
						Guilai (ST29)		
						Zigong (EX-CA1)		
						Xuehai (SP10)		
Lian et al, (2014)	Chinese	33/33	36 (2)/37 (3)	TEAS (day 1 of the ovarian stimulation cycle to the day of HCG, 30 min)/qod	mock TEAS (30 min)/qod	Guanyuan (RN4)	Not reported	①⑤
						Zhongji (RN3)		
						Zigong (EX-CA1)		
						Sanyinjiao (SP6)		
Xu et al, (2014)	Chinese	82/94	32.5 (4.6)/31.9 (4.3)	TEAS (the 10th day of menstruation to the day of ET, 100 Hz, 20–25 mA, and 30 min/qd; 24 h before ET and 30min after ET,2 Hz, 8–15 mA, and 30 min)	None	Tianshu (ST25)	Not reported	①②④
						Guanyuan (RN4)		
						Zhongji (RN3)		
						Zigong (EX-CA1)		
						Sanyinjiao (SP6)		
						Zusanli (ST36)		
						Taixi (KI3)		
						Shenshu (BL23)		
						Zhongwan (RN12)		
Shuai et al, (2015)	English	34/34	29.47 (3.24)/29.65 (2.60)	TEAS (3 menstrual cycles before FET, 2 Hz, 10–20 mA, 30 min/a total of 18 times)	mock TEAS (3 menstrual cycles before FET, intermittent 2 Hz, 5 mA, 30 min/a total of 18 times)	Zhongji (RN3)	Not reported	①②③
						Guanyuan (RN4)		
						Sanyinjiao (SP6)		
						Zigong (EX-CA1)		
Zheng et al, (2015)	English	56/56	36.05 (5.48)/36.88 (4.65)	TEAS (3 menstrual cycles before TVOR, 2 Hz, 20–25 mA, 30 min/qd)	comforting false Han’s placebo (3 menstrual cycles before TVOR, intermittent 5 mA, 30 min/qd)	Zhongji (RN3)	2 (mild allergy)/1(mild allergy)	①⑤
						Guanyuan (RN4)		
						Sanyinjiao (SP6)		
						Zigong (EX-CA1)		
						Tianshu (ST25)		
						Shenshu (BL23)		
						Yaoyangguan (DU3)		
						Mingmen (DU4)		
Zhong et al, (2016)	Chinese	50/50	31.37 (2.91)/33.19 (2.57)	TEAS (day 2–3 of the menstrual cycle t to the day of ET, 2 Hz, 8–25 mA, 30 min/qd)	mock TEAS (Day 2–3 of the menstrual cycle to before ET, invalid stimulus, 30 min/qd)	Guanyuan (RN4)	Not reported	①
						Zigong (EX-CA1)		
						Shenshu (BL23)		
						Sanyinjiao (SP6)		
Qu et al, (2017a)	English	108/109	31.22 (5.92)/29.81 (6.17)	TEAS (24 h before TVOR and 2 h before ET, 2 Hz,30 min)	None	Xuehai (SP10)	Not reported	①②③
						Diji (SP8)		
						Taichong (LR3)		
						Zusanli (ST36)		
						Zigong (EX-CA1)		
						Guanyuan (RN4)		
						Neiguan (PC6)		
						Zhongwan (RN12)		
Zhong and Zhong, (2017)	Chinese	735/1026	31 (4)/32 (4)	TEAS (24 h before ET and 2 h after ET, 2 Hz, 30 min)	None	Xuehai (SP10)	Not reported	①④
						Diji (SP8)		
						Zhongwan (RN12)		
						Guanyuan (RN4)		
						Zusanli (ST36)		
						Taixi (KI3)		
Zhang and Zhong, (2018)	Chinese	345/646	31.8 (4.1)/30.5 (4.3)	TEAS (24 h before ET and 2 h after ET, 2 Hz, 30 min; 2 days after ET, 2Hz, 30 min, qd/a total of 7 times)	None	Zigong (EX-CA1)	Not reported	①③
						Xuehai (SP10)		
						Diji (SP8)		
						Zhongwan (RN12)		
						Guanyuan (RN4)		
						Zusanli (ST36)		
						Taixi (KI3)		
						Neiguan (PC6)		
Dong et al, (2018)	Chinese	40/40	32.0 (5.0)/31.0 (4.0)	TEAS (day 2–3 of the menstrual cycle t to the day of ET, 2 Hz, 20–25 mA, and 30 min/qd) + Comparison	Ovulation induction medicine	Mingmen (DU4)	Not reported	①②④
						Shiqizhui (EX-B8)		
						Geshu (BL17)		
						Shenshu (BL23)		
						Taixi (KI3)		
						Sanyinjiao (SP6)		
						Zigong (EX-CA1)		
						Qihai (RN6)		
						Zusanli (ST36)		
						Guanyuan (RN4)		
						Fuliu (KI7)		
						Guanyuan (RN4)		
						Qihai (RN6)		
						Taichong (LR3)		
						Yanglingquan (GB34)		
Shuai et al, (2019)	English	61/61	31.23 (3.78)/31.58 (3.07)	TEAS (day 5 of the ovarian stimulation cycle to the day of ET, 2Hz, 9–25 mA, 30 min/qod)	mock TEAS (day 5 of the ovarian stimulation cycle to the day of ET, intermittent 2Hz and 5 mA, 30 min/qod)	Sanyinjiao (SP6)	Not reported	①②③
						Zhongji (RN3)		
						Guanyuan (RN4)		
						Zigong (EX-CA1)		
Shuai et al, (2019)	Chinese	30/30	35.9 (3.1)/36.1 (2.6)	TEAS (day 1 of the ovarian stimulation cycle to the day of HCG, 2 Hz, 20–25 mA,20min, 3 times/w)	None	Guanyuan (RN4)	Not reported	①⑤
						Tianshu (ST25)		
						Shenshu (BL23)		
						Mingmen (DU4)		
						Yaoyangguan (DU3)		
						Zigong (EX-CA1)		
						Sanyinjiao (SP6)		
Zhao and Fang, (2019)	Chinese	44/44	38.26 (2.48)/37.62 (2.27)	TEAS (day 2–3 of the menstrual cycle to the day of HCG, 30 min/qod)	mock TEAS (Day 2–3 of the menstrual cycle to the day of HCG, 30min/qod)	Zigong (EX-CA1)	Not reported	①⑤
						Sanyinjiao (SP6)		
						Guanyuan (RN4)		
						Zhongji (RN3)		
Fang et al, (2014)	Chinese	82/79	31.36 (3.37)/31.21 (3.37)	TEAS (two menstrual cycles before HCG to the day of HCG, 2 Hz, 20–25 mA,30min,/qd)	mock TEAS (two menstrual cycles before HCG to the day of HCG, intermittent invalid stimulus,30min,/qd)	Guanyuan (RN4)	Not reported	①②③⑤
						Zhongji (RN3)		
						Sanyinjiao (SP6)		
						Zigong (EX-CA1)		
						Tianshu (ST25)		
						Shenshu (BL23)		
						Yaoyangguan (DU3)		
						Mingmen (DU4)		
Zhai et al, (2022)	English	40/40	31.03 (3.06)/31.28 (3.56)	TEAS (day 3 of the menstrual cycle to the day of HCG, 30 mA, 30min/qd)	mock TEAS (day 3 of the menstrual cycle to the day of HCG, 5 mA, 30min/qd)	Zigong (EX-CA1)	Not reported	①
						Sanyinjiao (SP6)		
						Guanyuan (RN4)		
						Zhongji (RN3)		
						Taixi (KI3)		

I, intervention group; C, comparison group; h, hour; d, day; w, week; qd, once a day; qod, once every other day; TVOR, Trans-vaginal oocyte retrieval; HCG, HCG, injection day; ET, embryo transfer; FET, Frozen-thawed embryo transfer.

①, Clinical pregnancy rate; ②, Embryo implantation rate; ③, Live birth rate; ④, Biochemical pregnancy rate; ⑤, Number of oocytes retrieved.

### 3.3 risk of bias

**FIGURE 2:**
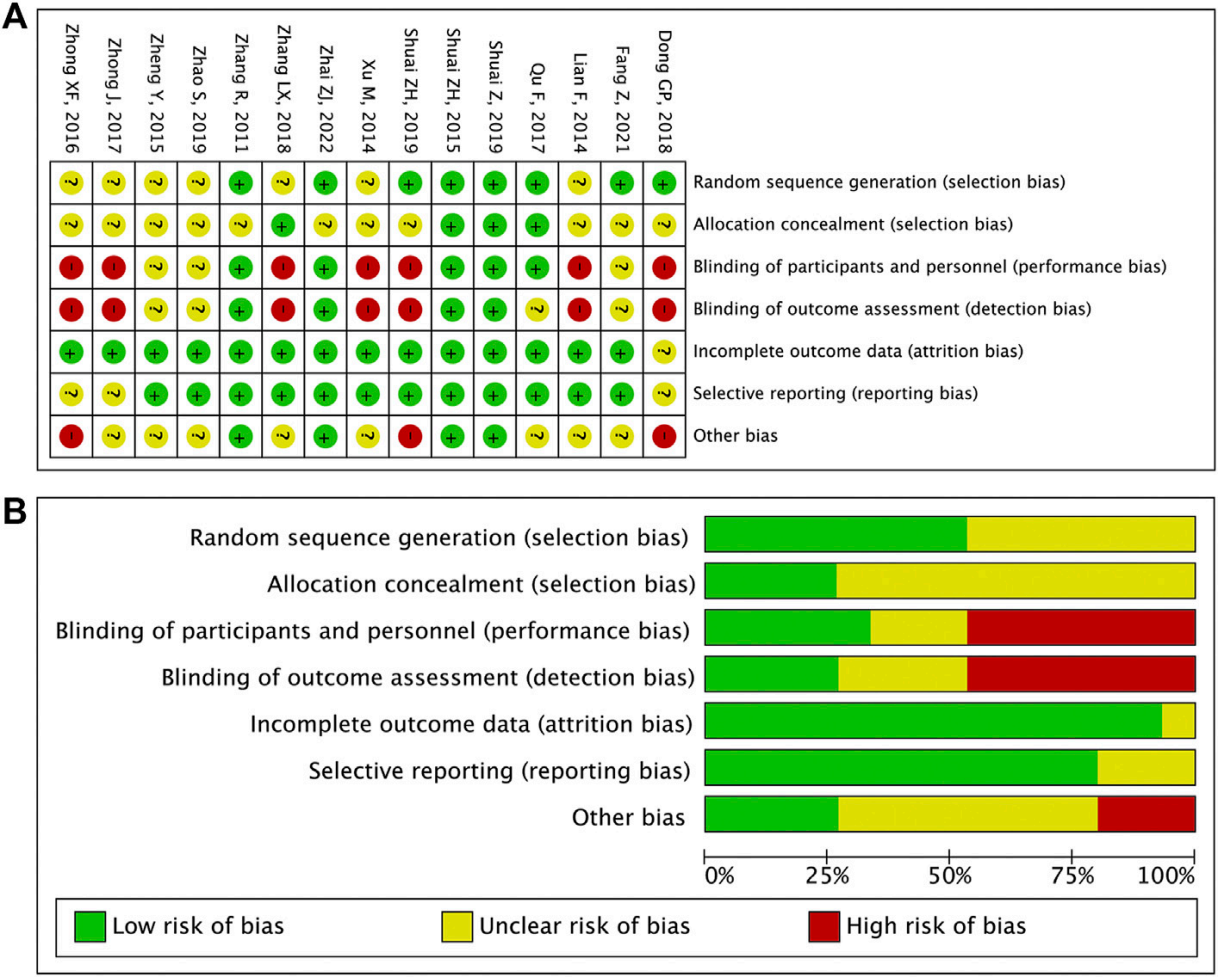
**(A)** Risk of bias item for included RCTs. **(B)** Risk of bias item presented as percentages across all included RCTs.

Eight RCTs (53.3%) were rated as low risk of bias due to random sequence generation. Four RCTs (26.7%) had a low risk of bias on allocation concealment. There was no study with a high risk of bias in terms of random sequence generation and allocation concealment. Five RCTs (33.3%) had a low risk of bias on blinding of participants and personnel. Four RCTs (26.7%) were rated as low risk of bias for blinding of the outcome assessment. Incomplete outcome data of 14 RCTs (93.3%) were rated as low risk of bias. Selective reporting of 12 RCTs (80.0%) were rated as low risk of bias. No study had a high risk of bias in incomplete outcome data and selective reporting. See Figure 2 for details.

### 3.4 Outcomes

#### 3.4.1 Primary outcomes

**FIGURE 3:**
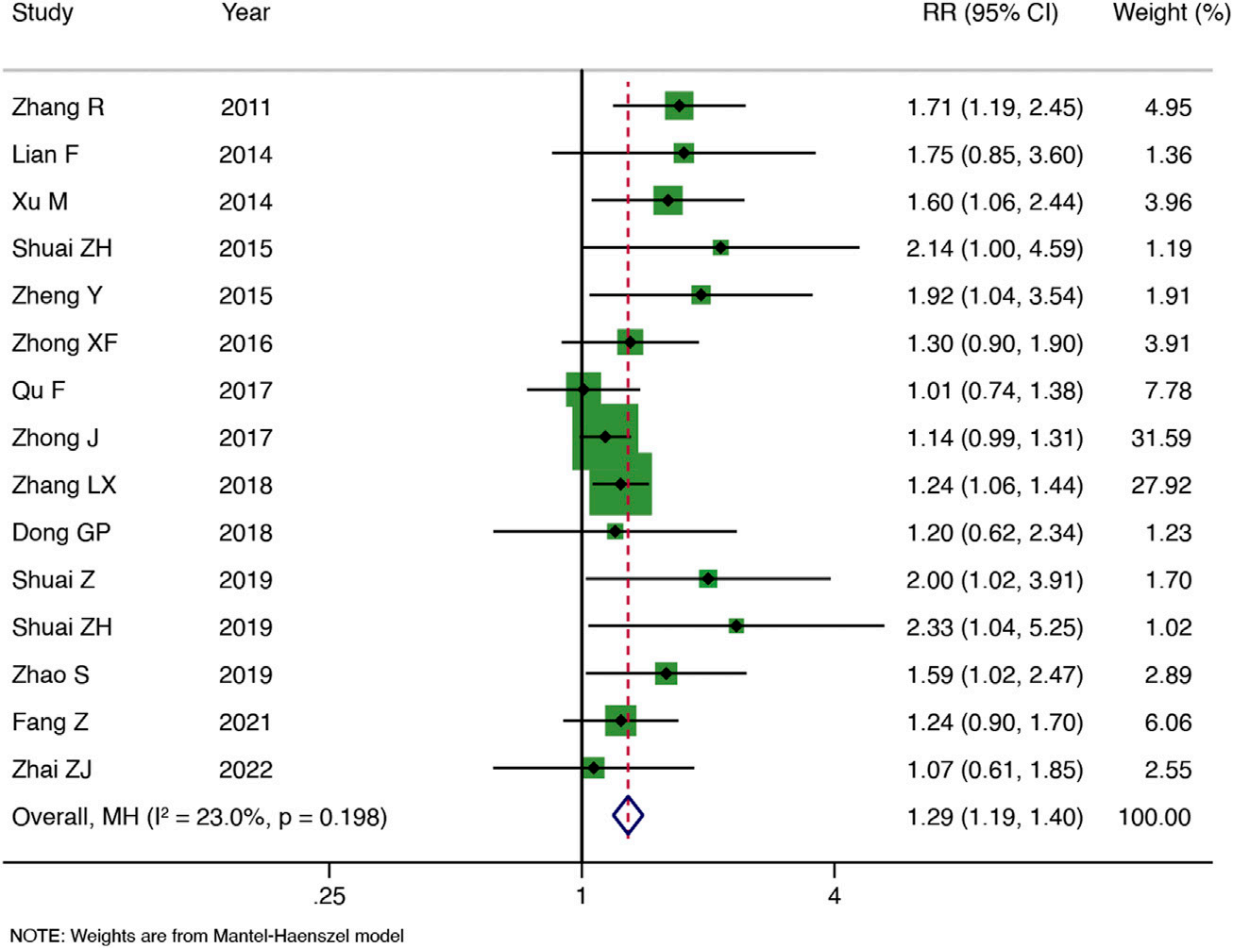
Meta-analysis of the clinical pregnancy rate.

The clinical pregnancy rate was reported in 15 RCTs (Figure 3). A meta-analysis showed that TEAS improved the clinical pregnancy rate by more in IVF-ET patients than in the control group [RR: 1.29, 95% CI: 1.19 to 1.40; *p* < 0.001; I^2^ = 23.0%], and the heterogeneity was low.

#### 3.4.2 Secondary outcomes

**FIGURE 4:**
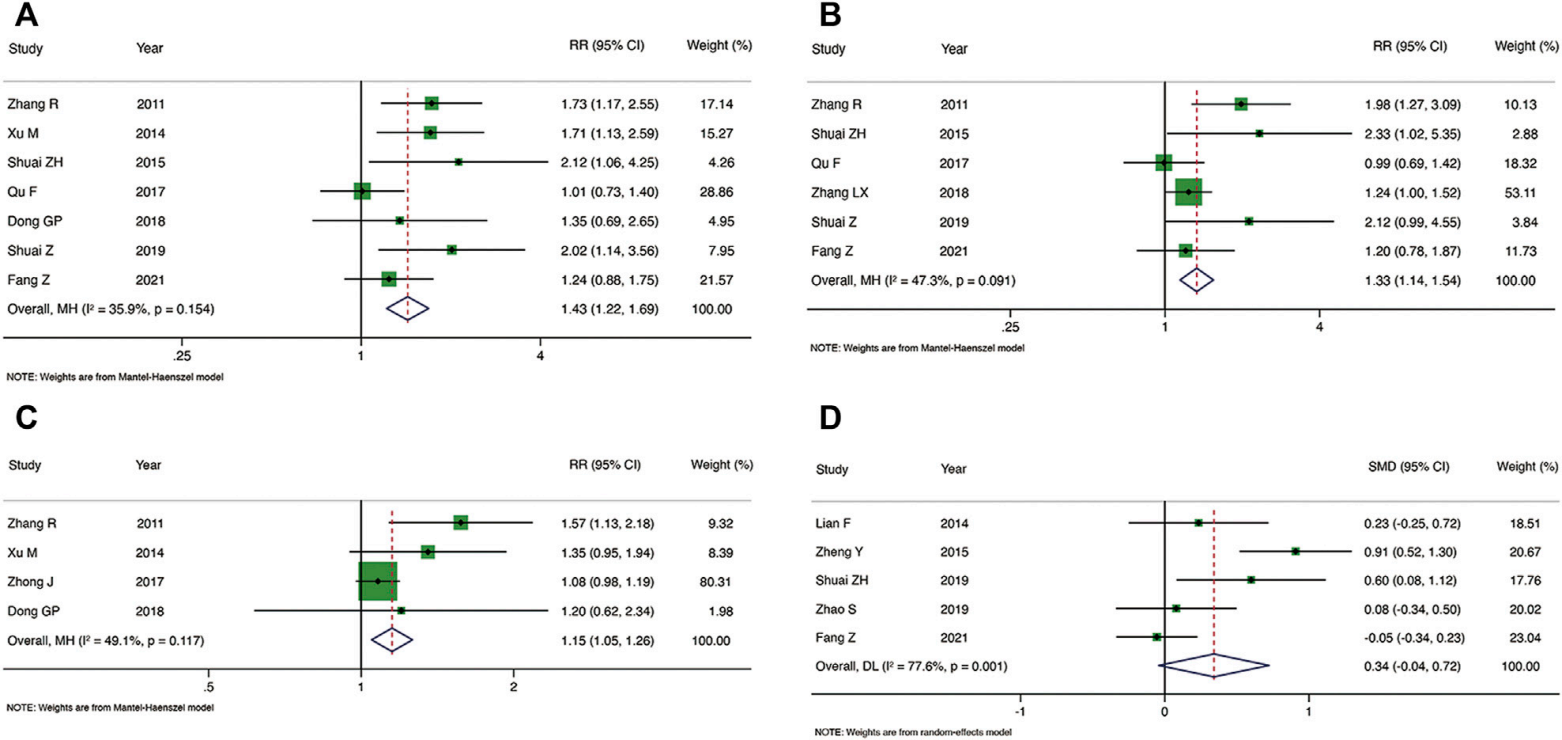
Meta-analysis of the embryo implantation rate **(A)**, live birth rate **(B)**, biochemical pregnancy rate **(C)** and the number of oocytes retrieved **(D)**.

Seven RCTs reported the embryo implantation rate (Figure 4A). As compared with the control group, TEAS increased the embryo implantation rate of IVF-ET patients, and the heterogeneity was low [RR: 1.43, 95% CI: 1.22 to 1.69; *p* < 0.001; I^2^ = 35.9%]. TEAS were found to be superior to the control group at improving the live birth rate in six RCTs [RR: 1.33, 95% CI: 1.14 to 1.54; *p* < 0.001; I^2^ = 47.3%] (Figure 4B). In addition, four RCTs reported the biochemical pregnancy rate, and TEAS showed the advantage of increasing the biochemical pregnancy rate in IVF-ET patients compared to the control group [RR: 1.15, 95% CI: 1.05 to 1.26; *p* = 0.003; I^2^ = 49.1%]) (Figure 4C). No significant heterogeneity was found among the studies. The results of five RCTs showed that the number of oocytes retrieved was not significantly greater in the TEAS vs. the control group, and the heterogeneity was high [SMD: 0.34, 95% CI: 0.04 to 0.72; *p* = 0.081; I^2^ = 77.6%] (Figure 4D).

### 3.5 Subgroup and sensitivity analysis resource identification initiative

We performed subgroup analysis of the clinical pregnancy rate, embryo implantation rate, and live birth rate according to the sample size, interventions and intervention time-point in the control group, see Table 2 for details. The results showed that among the above three outcomes, compared with the control group, whether the sample size was ≥100 or <100 had no effect on the results, and there was no obvious heterogeneity. Compared with ovulation-inducing medication, there was no obvious advantage of TEAS for improving the clinical pregnancy rate or the embryo implantation rate. Compared with no intervention, TEAS was not associated with a statistically significant increase in the embryo implantation rate or live birth rate and was accompanied by higher heterogeneity. Compared with the control group, TEAS at the intervention time-point of three menstrual cycles before FET or TVOR could better improve the clinical pregnancy rate, but the subgroup analysis of intervention time-point had no effect on the embryo implantation rate and live birth rate. Finally, the number of oocytes retrieved could only be divided into two subgroups based on intervention time-point (until the day of HCG and three menstrual cycles before FET or TVOR). The results showed until the day of HCG [4 RCTs, SMD: 0.16, 95% CI: −0.11 to 0.42; *p* = 0.245; I^2^ = 40.4%], three menstrual cycles before FET or TVOR [1 RCT, SMD: 0.91, 95% CI: 0.52 to 1.30; *p* < 0.001].

**TABLE 2:** Subgroup analyses of Clinical pregnancy rate, Embryo implantation rate and Live birth rate.

Outcomes	Clinical pregnancy rate			Embryo implantation rate			Live birth rate		
Subgroups	Studies	RR (95% CI)	I^2^ (*p*-value)	Studies	RR (95% CI)	I^2^ (*p*-value)	Studies	RR (95% CI)	I^2^ (*p*-value)
Overall analysis	15	1.29 (1.19, 1.40)	23.0% (*p* = 0.198)	7	1.43 (1.22, 1.69)	35.9% (*p* = 0.154)	6	1.33 (1.14, 1.54)	47.3% (*p* = 0.091)
Sample size of participants									
Participants <100	6	1.57 (1.22, 2.02)	0.0% (*p* = 0.526)	2	1.71 (1.05, 2.76)	0.0% (*p* = 0.356)	−		
Participants ≥100	9	1.26 (1.15, 1.37)	31.4% (*p* = 0.167)	5	1.41 (1.18, 1.67)	49.7% (*p* = 0.093)	−		
Comparison									
Mock TEAS	9	1.52 (1.30, 1.78)	0.0% (*p* = 0.601)	4	1.60 (1.28, 2.00)	14.8% (*p* = 0.318)	4	1.72 (1.31, 2.25)	19.6% (*p* = 0.292)
None	5	1.20 (1.09, 1.33)	37.1% (*p* = 0.174)	2	1.25 (0.97, 1.61) ^a^	74.0% (*p* = 0.050)	2	1.17 (0.98, 1.41) ^a^	7.2% (*p* = 0.299)
Ovulation induction medicine	1	1.20 (0.62, 2.34) ^a^	−	1	1.35 (0.69, 2.65) ^a^	−	−		
Intervention time-point									
24 h before ET and (or) 24 h after ET	8	1.24 (1.14, 1.36)	27.1% (*p* = 0.213)	5	1.45 (1.20, 1.75)	46.5% (*p* = 0.113)	4	1.31 (1.11, 1.54)	59.7% (*p* = 0.059)
Until the day of HCG	5	1.41 (1.14, 1.75)	0.0% (*p* = 0.443)	1	1.24 (0.88, 1.75) ^a^	−	1	1.20 (0.78, 1.87) ^a^	−
Three menstrual cycles before FET or TVOR	2	2.01 (1.25, 1.40)	0.0% (*p* = 0.828)	1	2.12 (1.06, 4.25) ^a^	−	1	2.33 (1.02, 5.35) ^a^	−

^a^ The result of the subgroup was not achieving statistical significance; RR, risk ratio; CI, confidence interval.

**FIGURE 5:**
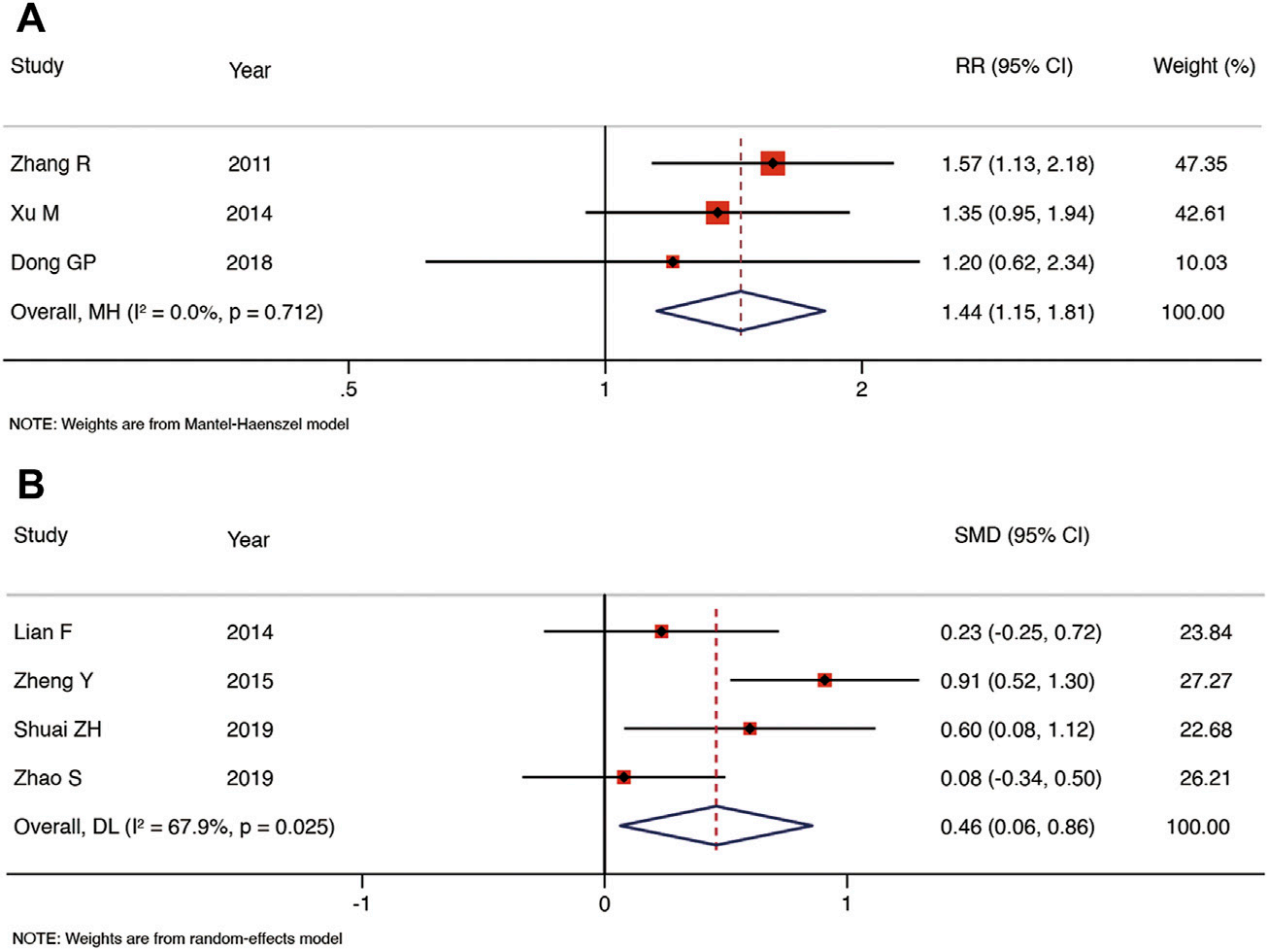
Meta-analysis of biochemical pregnancy rate **(A)** and the number of oocytes retrieved **(B)** after exclusion of studies.

We conducted a sensitivity analysis on each outcome, and the results showed that the clinical pregnancy rate, embryo implantation rate, and live birth rate were stable. The sensitivity analysis revealed that the biochemical pregnancy rate and number of oocytes retrieved were unstable. However, the heterogeneity of the biochemical pregnancy rate was significantly reduced following exclusion of Zhong et al. (Zhong and Zhang, 2017) [RR: 1.44, 95% CI: 1.15 to 1.81; *p* < 0.001; I^2^ = 0.0%] (Figure 5A). The number of oocytes retrieved was statistically significant, and the heterogeneity decreased following exclusion of Fang et al. (Zhou et al., 2021) [SMD: 0.46, 95% CI: 0.06 to 0.86; *p* = 0.023; I^2^ = 67.9%] (Figure 5B).

### 3.6 Publication bias

The clinical pregnancy rates from more than 10 studies were assessed for publication bias, and both Begg’s test and Egger’s test indicated publication bias (Supplementary Materials). The results were stable following modification by the trim-and-fill method.

### 3.7 GRADE

Due to the high risk of bias and publication bias, the quality of evidence was rated as low for the clinical pregnancy rate, embryo implantation rate, live birth rate, and biochemical pregnancy rate. In addition to the above reasons, the number of oocytes retrieved was rated as very low due to high heterogeneity, as detailed in Supplementary Materials.

## 4 Conclusion

### 4.1 Summary of main results

The pooled results showed that TEAS improved the rates of clinical pregnancy, embryo implantation, live birth, and biochemical pregnancy in IVF-ET patients. TEAS improved the number of oocytes retrieved significantly following exclusion of one study. TEAS and ovulation-inducing medication did not significantly improve the clinical pregnancy rate. However, only one study used ovulation-inducing medication as a control. Subgroup analysis showed that sample size had no effect on the results. The original study presented limited safety data, and the main results of this review had a publication bias and low evidence quality.

TEAS evolved from acupuncture techniques. In contrast to traditional acupuncture, TEAS is a non-invasive electrical stimulation technique that avoids infection caused by needles piercing the skin, reduces patient fear (Zhang et al., 2014), and combines the advantages of percutaneous nerve electrical stimulation and acupoint stimulation (Szmit et al., 2021). TEAS differs from electroacupuncture in that electroacupuncture acts directly on precise acupoints, while TEAS uses electrodes to expand the stimulation range, which includes the skin in the vicinity of the acupoints (Chen et al., 2022). TEAS has been used for assisted reproduction (Hsu et al., 2017; Shuai et al., 2019) and the treatment of asthenozoospermia (Yu et al., 2019; Jin et al., 2021), labor pain (Dowswell et al., 2009; Bedwell et al., 2011; Liu et al., 2015), and dysmenorrhea (Lewers et al., 1989; Wu et al., 2012).

In 2002, Paulus et al. (2002) first reported that acupuncture could improve the clinical pregnancy rate in patients undergoing assisted reproduction. However, with the continuous development of ART, improving the clinical pregnancy rate has been a challenge in the field of assisted reproduction. Fertility specialists have proposed various ovarian stimulation options to improve IVF-ET pregnancy outcomes. However, the quality of evidence supporting these interventions remains controversial. Over the past 10 years, TEAS has been widely used in assisted reproduction, but its clinical efficacy is not yet clear. This review evaluated the efficacy and safety of TEAS for improving pregnancy outcomes in women undergoing IVF-ET. These outcomes were similar to those of previous research (Zhan et al., 2021); however, this review provided a more comprehensive assessment. As the primary outcome of the systematic review, the clinical pregnancy rate in the TEAS group was significantly greater than that of the control group, there was no obvious heterogeneity, and the sensitivity analysis was also stable. TEAS also significantly improved the embryo implantation and live birth rates. We further examined the sources of heterogeneity and found that following the exclusion of one study, the heterogeneity of the biochemical pregnancy rate decreased significantly. Compared with the control group, the number of oocytes retrieved in the TEAS group was statistically significant, and the heterogeneity was decreased following the exclusion of one study. In this review, a total of 7 RCTs reported the effects of TEAS on embryo quality. Due to the small amount of studies and the differences in intervention details, we conducted a descriptive analysis based on the results. 5 RCTs (Fang et al., 2014; Dong et al., 2018; Zhao and Fang, 2019; Zhenhong and Yang, 2019; Zhou et al., 2021) showed that TEAS was able to improve the high-quality embryo rates compared to the control group. However, the other two studies (Zheng et al., 2015; Qu et al., 2017b) found no significant differences. Further evidence is needed to confirm whether TEAS improve embryo quality.

Previous studies have found that mTEAS may improve pregnancy outcomes somewhat, but this finding may be due to the placebo effect of routine procedures; it may also indicate that a weak current or electrodes without electricity are not completely inert (Zhuo et al., 2022). An adequate literature search was conducted for this review, but RCTs were few, and the sample size was insufficient. We therefore conclude that there is limited evidence supporting the safety and effectiveness of TEAS for improving pregnancy outcomes in women undergoing IVF-ET. Future studies will require improved study designs, expanded sample sizes, and a higher quality of evidence to verify our conclusions.

The 15 RCTs included a total of 21 acupoints, and the top five most frequently used acupoints were Guanyuan (RN4, 15 times), Zigong (EX-CA1, 14 times), Sanyinjiao (SP6, 11 times), Zhongji (RN3, 8 times), and Shenshu (BL23, 6 times). See the Supplementary Materials for details. In modern reproductive medicine, embryo implantation is a process of uterine implantation *via* the interaction between the blastocyst and the endometrium. However, blastocyst implantation disorder is one of the main causes of pregnancy failure, and embryo quality and developmental potential directly affect the pregnancy outcome. The theory of Traditional Chinese Medicine (TCM) purports that the sperm-ovum-embryo axis in assisted reproductive technology is closely related to the essence of the kidney. IVF-ET patients have often undergone long-term use of exogenous hormones to promote ovulation or because of poor functional status, resulting in a deficiency of kidney essence. TCM mainly focuses on tonifying the kidney and improving its essence. RN4 and RN3 belong to Ren Meridian, both located on the abdomen, and are useful for treating diseases of the reproductive system. SP6 belongs to the spleen Meridian of Foot-Taiyin, which mainly treats gynecological and obstetric diseases such as irregular menstruation, dysmenorrhea, and infertility. BL23 is the acupoint of the Bladder Meridian of Foot-Taiyang, cooperating with EX-CA1 to improve the patient’s uterine environment and help the embryo to implant smoothly. The above acupoints are effective for the treatment of infertility (Wang et al., 2010). TEAS take acupoints as a starting point and exerts curative effects by means of electrical nerve stimulation, which is the organic combination of syndrome differentiation and the treatment of TCM and modern science and technology.

### 4.2 Strengths and limitations

Clarifying the ability of TEAS to improve the efficacy and safety of IVF-ET pregnancy outcomes through systematic evaluation and meta-analysis is critical for clinical decision making. This review protocol has been pre-registered on PROSPERO and has been reported in the literature, in strict compliance with the PRISMA statement. Secondly, we focused on several of the most important clinical outcomes in IVF-ET, such as the rates of clinical pregnancy, live birth, and embryo implantation, and clarified the measurement criteria for these indicators, rather than focusing on the quantity and quality of follicles, thus providing data conducive to evidence-based medicine. Finally, we performed a subgroup analysis to seek sources of heterogeneity, which added stability to the results. However, there are some limitations to this review. The experimental groups differed in acupoint selection and the frequency and course of treatment, and the number of RCTs was limited. We were unable to conduct subgroup analysis for these potential influencing factors, which may have increased the heterogeneity of the results and reduced the quality of evidence. In addition, the sample sizes of the original studies varied greatly, and most of them were single-center trials, which may have led to publication bias. Similarly, adverse events were rarely reported in the original studies, and safety issues cannot be guaranteed. Therefore, we have interpreted the results with caution.

## 5 Conclusion

Current evidence supports the potential clinical value of TEAS as an adjuvant therapy in assisted reproduction. TEAS has positive effects on the clinical pregnancy rate, embryo implantation rate, live birth rate, biochemical pregnancy rate, and number of oocytes retrieved. However, considering the low quality and publication bias of present studies, a cautious and conservative recommendation for broader clinical use of TEAS should still be made, further high-quality, large-sample RCTs are necessary to demonstrate that TEAS improves the efficacy and safety of IVF-ET pregnancy outcomes in women.
