# Supplementary material for: Effect of transcutaneous electrical acupoint stimulation on pregnancy outcomes in women with in vitro fertilization-embryo transfer: A systematic review and meta-analysis
Source: Front Cell Dev Biol. 2022 Dec 12;10:1068894. doi: 10.3389/fcell.2022.1068894 (PMC9791369; doi:10.3389/fcell.2022.1068894)
Supplement: Supplementary file 2 [file DataSheet1.PDF]

Table S1 Search strategy

PubMed

#1 ((((transcutaneous electrical acupoint stimulation) OR (TEAS)) OR (transcutaneous acupoint electrical stimulation)) OR (TAES)) OR (acustimulation)

#2 ((((((((fertilizations in vitro[MeSH Terms]) OR (in vitro fertilization[Text Word])) OR (IVF[Text Word])) OR (embryo transfer[Text Word])) OR (ET[Text Word])) OR (in vitro fertilization-embryo transfer[Text Word])) OR (IVF-ET[Text Word])) OR (test-tube fertilization[Text Word])) OR (test-tube baby[Text Word])

#3 ((clinical trial) OR (randomized controlled trial)) OR (trials)

#4 #1 and #2 and #3

Cochrane library

#1 (transcutaneous electrical acupoint stimulation):ti,ab,kw

#2 TEAS:ti,ab,kw

#3 transcutaneous acupoint electrical stimulation:ti,ab,kw

#4 TAES:ti,ab,kw

#5 acustimulation:ti,ab,kw

#6 #1 or #2 or #3 or #4 or #5 1

#7 fertilizations in vitro:MeSH

#8 in vitro fertilization:ti,ab,kw

#9 IVF:ti,ab,kw

#10 embryo transfer:ti,ab,kw

#11 ET:ti,ab,kw

#12 in vitro fertilization-embryo transfer:ti,ab,kw

#13 IVF-ET:ti,ab,kw

#14 test-tube fertilization:ti,ab,kw

#15 test-tube baby:ti,ab,kw

#16 #7 or #8 or #9 or #10 or #11 or #12 or #13 or #14 or #15

#17 clinical trial:ti,ab,kw

#18 randomized controlled trial:ti,ab,kw

#19 trials:ti,ab,kw

#20 #17 or #18 or #19

#21 #6 and #16 and #20

Embase

#1 'transcutaneous electrical acupoint stimulation'/exp OR 'transcutaneous electrical acupoint stimulation' OR (transcutaneous AND electrical AND ('acupoint'/exp OR acupoint) AND ('stimulation'/exp OR stimulation)) OR teas OR (transcutaneous AND acupoint AND electrical AND stimulation)
OR taes OR acustimulation

#2 'fertilizations in vitro' OR (fertilizations AND in AND vitro) OR 'in vitro fertilization' OR ivf OR (embryo AND transfer) OR et OR (in AND vitro AND 'fertilization embryo' AND transfer) OR 'ivf et' OR ('test tube' AND fertilization)

#3 'clinical trial'/exp OR 'clinical trial' OR (('clinical'/exp OR clinical) AND ('trial'/exp OR trial)) OR (randomized AND controlled AND trial) OR trials

#4 #1 AND #2 AND #3

Web of science

#1 ((((ALL=(transcutaneous electrical acupoint stimulation)) OR ALL=(TEAS)) OR ALL=(transcutaneous acupoint electrical stimulation)) OR ALL=(TAES)) OR ALL=(acustimulation)

#2 ((((((((ALL=(fertilizations in vitro)) OR ALL=(in vitro fertilization)) OR ALL=(IVF)) OR ALL=(embryo transfer)) OR ALL=(ET)) OR ALL=(in vitro fertilization-embryo transfer)) OR ALL=(IVF-ET)) OR ALL=(test-tube fertilization)) OR ALL=(test-tube fertilization)

#3 ((ALL=(clinical trial)) OR ALL=(randomized controlled trial)) OR ALL=(trials)

#4 #1 AND #2 AND #3

CNKI

SU=('经皮穴位电刺激'+经皮电刺激'+'TEAS+'TAES') AND SU=('体外授精-胚胎移植'+'体外授精'+'胚胎移植'+'辅助生殖'+'IVF-ET') AND SU= ('随机对照试验'+'临床研究'+'临床试验'+'临床观察'+'疗效'+'影响')

SinoMed

1) "经皮穴位电刺激"[常用字段:智能] OR "经皮电刺激"[常用字段:智能] OR "TEAS"[常用字段:智能] OR "TAES"[常用字段:智能]

2) "体外授精-胚胎移植"[常用字段:智能] OR "体外授精"[常用字段:智能] OR "胚胎移植"[常用字段:智能] OR "辅助生殖"[常用字段:智能] OR "IVF-ET"[常用字段:智能]

3) "随机对照试验"[常用字段:智能] OR "临床研究"[常用字段:智能] OR "临床试验"[常用字段:智能] OR "临床观察"[常用字段:智能] OR "疗效"[常用字段:智能] AND "影响"[常用字段:智能]

4) ("随机对照试验"[常用字段:智能] OR "临床研究"[常用字段:智能] OR "临床试验"[常用字段:智能] OR "临床观察"[常用字段:智能] OR "疗效"[常用字段:智能] AND "影响"[常用字段:智能]) AND ("体外授精-胚胎移植"[常用字段:智能] OR "体外授精"[常用字段:智能] OR "胚胎移植"[常用字段:智能] OR "辅助生殖"[常用字段:智能] OR "IVF-ET"[常用字段:智能]) AND ("经皮穴位电刺激"[常用字段:智能] OR "经皮电刺激"[常用字段:智能] OR "TEAS"[常用字段:智能] OR "TAES"[常用字段:智能])

Table S2 Exclusion list

Reference	Reason
Hsu YC, Liang IT, Huang SY, Wang HS, Soong YK, Chang CL. Transcutaneous electrical acupoint stimulation (TEAS) treatment improves pregnancy rate and implantation rate in patients with implantation failure. Taiwan J Obstet Gynecol. 2017 Oct;56(5):672-676.	Not RCT
Zhang R, Li R, Feng XJ, Han SP, Sun W, Qiao J, et, al. Transcutaneous electrical acupoint stimulation improves clinical pregnancy rate in embryo transfer. Human Reproduction. 2014, 29:i365.	Conference Abstract
Qu F.Transcutaneous electrical acupoint stimulation alleviates the anxiety levels of IVF and improves the outcomes: a prospective, randomized and controlled study. Advances in Integrative Medicine. 2019,6:S28.	Conference Abstract
Zhong XF, Jiang XM, Yin P, Yan H, Heng BC, Zhang WW, et, al. Transcutaneous electrical acupoint stimulation (TEAS) improves IVF outcome in patients receiving vitrified-warmed embryo-transfer.Journal of Investigative Medicine.2019, 67:A5.	Conference Abstract
Zhou Y.Effect of percutaneous electrical acupoint stimulation on plasma levels of oxytocin and vasopressin on pregnancy outcome in patients with anxiety and depression [D]. Shandong University of Chinese Medicine,2016.	Participants are not satisfied
Zhang J, Zhou YX, Tao SH, Xie D, Xu Y, Wang LQ,et al. Effects of percutaneous electrical acupoint stimulation and low dose of chorionic gonadotropin on pregnancy outcome of freeze-thaw embryo transfer [J]. Chinese journal of integrated traditional and western medicine,2019,39(10):1177-1180.	Retrospective analysis

Table S3 GRADE Table.

Outcomes	Certainty assessment					Quality of evidence
	Risk of bias	Inconsistency	Indirectness	Imprecision	Publication bias	
Clinical pregnancy rate	Serious[a]	Not serious	Not serious	Not serious	Serious[c]	Low
Embryo implantation rate	Serious[a]	Not serious	Not serious	Not serious	Serious[c]	Low
Live birth rate	Serious[a]	Not serious	Not serious	Not serious	Serious[c]	Low
Biochemical pregnancy rate	Serious[a]	Not serious	Not serious	Not serious	Serious[c]	Low
Number of oocytes retrieved	Serious[a]	Serious[b]	Not serious	Not serious	Serious[c]	Very Low

GRADE, Grading of Recommendations Assessment, Development, and Evaluation.

[a] *The risk of bias is unclear in most of the studies.*

[b] *The confidence interval overlap less, the heterogeneity test P was very small, and the I2 was larger (I^2 threshold value: 50%).*

[c] *Funnel plot was not symmetrical, or the number of included studies was small and all were positive results.*

Table S4 Frequency of acupuncture points

Acupounts	Frequency	Meridian
Guanyuan (RN4)	15 times	Ren Meridian
Zigong (EX-CA1)	14 times	Extra Nerve Points
Sanyinjiao (SP6)	11 times	the Spleen Meridian of Foot-Taiyin
Zhongji (RN3)	8 times	Ren Meridian
Shenshu (BL23)	6 times	the Bladder Meridian of Foot-Taiyang
Zusanli (ST36)	5 times	the Stomach Meridian of Foot-Yangming
Taixi (KI3)	5 times	the Kidney Meridian of Foot-Shaoyin
Diji (SP8)	4 times	the Spleen Meridian of Foot-Taiyin
Xuehai (SP10)	4 times	the Spleen Meridian of Foot-Taiyin
Tianshu (ST25)	4 times	the Stomach Meridian of Foot-Yangming
Zhongwan (RN12)	4 times	Ren Meridian
Mingmen (DU4)	4 times	Governor Meridian
Yaoyangguan (DU3)	3 times	Governor Meridian
Qihai (RN6)	2 times	Ren Meridian
Taichong (LR3)	2 times	The Liver Meridian of Foot- Jueyin
Neiguan (PC6)	2 times	the Pericardium Meridian of Hand-Jueyin
Shiqizhui (EX-B8)	1 times	Extra Nerve Points
Guilai (ST29)	1 times	the Stomach Meridian of Foot-Yangming
Geshu (BL17)	1 times	the Bladder Meridian of Foot-Taiyang
Fuliu (KI7)	1 times	the Kidney Meridian of Foot-Shaoyin
Yanglingquan (GB34)	1 times	the Gallbladder Meridian of Foot-Shaoyang

Figure S1 Begg's test and Egger's test.

Begg's Test

```
  adj. Kendall's Score (P-Q) =      51
          Std. Dev. of Score =   20.21
           Number of Studies =      15
                          z  =    2.52
                    Pr > |z| =   0.012
                          z  =    2.47 (continuity corrected)
                    Pr > |z| =   0.013 (continuity corrected)
```

Egger's test

Std_Eff	Coef.	Std. Err.	t	P>|t|	[95% Conf.	Interval]
slope	.0471623	.0646388	0.73	0.479	-.0924814	.186806
bias	1.406754	.4052316	3.47	0.004	.531304	2.282203
